# Influence of Crohn’s disease related polymorphisms in innate immune function on ileal microbiome

**DOI:** 10.1371/journal.pone.0213108

**Published:** 2019-02-28

**Authors:** Ellen Li, Yuanhao Zhang, Xinyu Tian, Xuefeng Wang, Grace Gathungu, Ashley Wolber, Shehzad S. Shiekh, R. Balfour Sartor, Nicholas O. Davidson, Matthew A. Ciorba, Wei Zhu, Leah M. Nelson, Charles E. Robertson, Daniel N. Frank

**Affiliations:** 1 Department of Medicine, Stony Brook University, Stony Brook, NY, United States of America; 2 Department of Biostatistics and Bioinformatics, H. Lee Moffitt Cancer Center & Research Institute, Tampa, FL, United States of America; 3 Department of Pediatrics, Stony Brook University, Stony Brook, NY, United States of America; 4 Department of Medicine, University of North Carolina, Chapel Hill, NC, United States of America; 5 Department of Medicine, Washington University St. Louis, St. Louis, MO, United States of America; 6 Department of Applied Mathematics and Statistics, Stony Brook University, Stony Brook, NY, United States of America; 7 Department of Medicine, University of Colorado Anschutz Medical Campus, Aurora, CO, United States of America; Cincinnati Children's Hospital Medical Center, UNITED STATES

## Abstract

We have previously identified NOD2 genotype and inflammatory bowel diseases (IBD) phenotype, as associated with shifts in the ileal microbiome (“dysbiosis”) in a patient cohort. Here we report an integrative analysis of an expanded number of Crohn's disease (CD) related genetic defects in innate immune function (NOD2, ATG16L1, IRGM, CARD9, XBP1, ORMDL3) and composition of the ileal microbiome by combining the initial patient cohort (Batch 1, 2005–2010, n = 165) with a second consecutive patient cohort (Batch 2, 2010–2012, n = 118). These combined patient cohorts were composed of three non-overlapping phenotypes: 1.) 106 ileal CD subjects undergoing initial ileocolic resection for diseased ileum, 2.) 88 IBD colitis subjects without ileal disease (predominantly ulcerative colitis but also Crohn’s colitis and indeterminate colitis, and 3.) 89 non-IBD subjects. Significant differences (FDR < 0.05) in microbiota were observed between macroscopically disease unaffected and affected regions of resected ileum in ileal CD patients. Accordingly, analysis of the effects of genetic and clinical factors were restricted to disease unaffected regions of the ileum. Beta-diversity differed across the three disease categories by PERMANOVA (p < 0.001), whereas no significant differences in alpha diversity were noted. Using negative binomial models, we confirmed significant effects of IBD phenotype, *C*. *difficile* infection, and NOD2 genotype on ileal dysbiosis in the expanded analysis. The relative abundance of the Proteobacteria phylum was positively associated with ileal CD and colitis phenotypes, but negatively associated with NOD2^R^ genotype. Additional associations with ORMDL3 and XBP1 were detected at the phylum/subphylum level. IBD medications, such as immunomodulators and anti-TNFα agents, may have a beneficial effect on reversing dysbiosis associated with the IBD phenotype. Exploratory analysis comparing microbial composition of the disease unaffected region of the resected ileum between 27 ileal CD patients who subsequently developed endoscopic recurrence within 6–12 months versus 34 patients who did not, suggested that microbial biomarkers in the resected specimen helped stratify patients with respect to risk of post-surgical recurrence.

## Introduction

Inflammatory bowel disease (IBD) describes a group of disorders in which the intestines become inflamed. Two major types of IBD are ulcerative colitis (UC) and Crohn's disease (CD). UC is limited to the colon or large intestine. Crohn's disease, on the other hand, can involve any part of the gastrointestinal tract from the mouth to the anus. Most commonly, though, it affects the ileum or the colon or both. Abnormal host-microbial interactions and genetic susceptibility are implicated in the pathogenesis of IBD, reviewed in [1]. We previously examined the effects of the NOD2 and ATG16L1 polymorphisms on ileal microbial composition in 1) ileal CD subjects undergoing initial ileal resection, 2) IBD colitis without ileitis (predominantly ulcerative colitis) subjects and 3) subjects without inflammatory bowel disease (non-IBD) [2–4]. These previous studies identified NOD2 genotype and *C*. *difficile* infection, in addition to IBD phenotype, as associated with shifts in ileal microbiota or “dysbiosis” [2–4]. We also found that differential expression of genes involved in Paneth cell function were associated with shifts in ileal microbial composition [5]. In the current study, the effects of an expanded panel of Crohn’s disease risk alleles [6–10] that are associated with defects in innate immunity (NOD2, ATG16L1, IRGM, CARD9, XBP1, ORMDL3) on ileal microbial composition, were analyzed by combining the original patient cohort (referred to as “Batch 1 2005–2010”) with a second consecutive patient cohort collected between 2010 and 2012 (“Batch 2 2010–2012”, see **Table 1**”) Some of these genes are implicated in autophagy (e.g. ATG16L1, IRGM), or endoplasmic reticulum stress (e.g. XBP1, ORMDL3), and/or Paneth cell dysfunction (NOD2 ATG16L1, IRGM).

**Table 1:** Distribution of NOD2, ATG16L1, IRGM, CARD9 and ORMDL3 risk alleles and clinical characteristics in ileal CD, colitis and non-IBD subjects in batches 1 and 2. The percent of subjects recruited at each of the three IBD centers with complete genotype and clinical characteristics with at least one risk allele and the percent of subjects who have at least one risk allele (see Methods) are listed.

	Ileal CD			Colitis			Non-IBD		
	n = 106			n = 88			n = 89		
	Batch1	Batch2	P value	Batch1	Batch2	P value	Batch1	Batch2	P value
	n = 50	n = 56		n = 59	n = 29		n = 56	n = 33	
***IBD Center***									
Wash. U.-St. Louis	100%	62%		100%	83%		100%	73%	
Stony Brook U.	0%	9%		0%	17%		0%	27%	
U. of North Carolina	0%	29%		0%	0%		0%	0%	
***IBD Risk Allele***									
NOD2^R^ composite	40%	30%	0.38	19%	14%	0.78	14%	52%	<0.001
ATG16L1^R^	94%	79%	0.05	81%	76%	0.79	77%	79%	0.99
IRGM^R^	30%	27%	0.90	29%	17%	0.34	27%	18%	0.38
CARD9^R^	94%	96%	0.98	93%	93%	1	88%	91%	0.85
XBP1^R^	12%	14%	0.99	10%	14%	0.84	14%	0%	0.01
ORMDL3^R^	76%	73%	0.90	75%	79%	0.88	62%	70%	0.51
***Clinical covariates***									
Male gender %	46%	43%	0.91	53%	55%	1	41%	39%	0.99
Caucasian race %	94%	82%	0.11	91%	97%	0.55	89%	94%	0.56
Median age years (range)	33 (18–72)	32 (17–67)		44 (18–69)	47 (20–72)		61 (24–84)	62 (17–86)	
Duration IBD years (range)	5 (0–38)	5 (0–35)		5 (0–45)	5 (0.1–30)		NA	NA	
Current smoker %	32%	29%	0.90	10%	3%	0.46	27%	18%	0.38
+ fecal C. difficile toxin %	0%	5%	0.32	24%	0%	0.01	0%	6%	0.23
Colon Cancer %	6%	0%	0.20	17%	3%	0.13	54%	45%	0.46
Median BMI kg/m2 (range)	24 (16–41)	23 (14–44)		26 (16–43)	28 (19–36)		28 (18–47)	26 (20–48)	
5-ASA %	60%	38%	0.04	63%	48%	0.27	0%	3%	0.65
Steroids %	48%	54%	0.67	54%	52%	1	2%	12%	0.11
Immunomodulators %	48%	30%	0.09	29%	28%	1	4%	6%	0.98
Anti-TNF alpha %	24%	45%	0.04	31%	52%	0.09	2%	0%	0.96

The majority of patients with the ileal CD phenotype eventually undergo surgical resection of diseased ileum because of stricturing and penetrating complications [11]. Unfortunately, disease recurrence in previously disease-free segments of the ileum at the surgical anastomosis is common [12–16]. A reduced relative abundance of *Faecalibacterium prausnitzii*, a commensal anaerobic bacterium in the distal intestine (ileum and colon), has been a consistent feature associated with the ileal CD phenotype [1, 3,4,17–21]. This bacterial species, along with other closely related clostridial species, are key sources of the short chain fatty acid butyrate, which is the preferred energy source for enterocytes in the distal intestine, and exhibits anti-inflammatory and pro-intestinal barrier properties in experimental mouse models [22]. A diminished relative abundance of ileal *F*. *prausnitzii* at the time of resection has been associated with a higher risk of post-operative endoscopic recurrence of ileal CD six months after surgery [17,23–25]. Consequently, an additional goal of this study was to determine whether alterations in the relative abundances of specific bacterial taxa, such as *F*. *prausnitzii*, at the time of ileal resection were predictive of subsequent endoscopic recurrence. To this end, ileal microbiota in the disease unaffected region of the ileal resection were compared between a group of ileal CD subjects who subsequently developed endoscopic recurrence in the neo-terminal ileum and a group of ileal CD subjects who did not, 6–12 months after initial surgery.

## Materials and methods

### Patient recruitment

This study was approved by the Institutional Review Boards of Stony Brook University (IRB# 245010), Washington University-St. Louis (IRB# 201101774), and the University of North Carolina (IRB# 10–0355). Patient written consents were obtained from all study participants and assent and parental consents were obtained for children <18 years of age at their respective institutions. Coded samples stripped of all identifying information were collected from subjects in the three following categories: 1) Ileal CD patients undergoing initial ileocolic resection (ICR) of diseased ileum; 2) colitis patients without ileitis (predominantly ulcerative colitis or UC, but also Crohn’s colitis and indeterminate colitis) undergoing initial total colectomy; and 3) patients without IBD undergoing initial right or total colectomy were prospectively enrolled to donate tissue, blood and longitudinal clinical information in a consecutive fashion by the Stony Brook University GI Biobank (Batch 2, 2010–2012), the Washington University Digestive Diseases Research Core Center Biobank Core (Batch 1, 2005–2010; Batch 2, 2010–2012) and the U. of North Carolina Multidisciplinary IBD Center (Batch 2, 2011–2012) as previously described [4]. The diagnosis of ileal CD, UC, indeterminate colitis and Crohn’s colitis was made ultimately on the basis of pathological criteria (surgical resection specimen) [26–28]. A minimum of 4 ex-vivo biopsies were taken separately from the macroscopically disease unaffected proximal ileal margin (from all subjects) and from the disease affected region (from ileal CD subjects) of fresh pathological specimens using Radial Jaw4 large capacity biopsy forceps (Boston Scientific, Natick, MA), and immediately placed in RNA stabilization solution (RNAlater, Life Technologies, Grand Island, NY, USA) overnight at 4°C prior to storing at -80^o^ C. A subset of the ileal CD patients (n = 61) underwent follow up colonoscopy within 6–12 months to assess endoscopic disease recurrence as previously described [29]. *Ex-vivo* research ileal biopsies, blood and longitudinal clinical information were also prospectively collected from non-IBD patients undergoing colonoscopies for colon cancer screening by the Stony Brook University GI Biobank (2010–2012) and the Washington University Digestive Diseases Research Core Center Biobank Core (2005–2012).

Additional clinical metadata on smoking, obesity, and IBD medications were obtained on samples collected by the Stony Brook University GI Biobank and the Washington University Digestive Disease Research Core Center Biobank Core and Dr. Sartor’s laboratory, by reviewing the medical records including the pathology report of the resected intestine by a gastroenterologist (EL, RBS) [4]. Preoperative mechanical bowel preparations were not routinely ordered for surgical procedures on IBD patients but were often ordered for the non-IBD patients. A smoker was defined as smoking ≥7 cigarettes a week for at least a year [12,30]. In order to assess the potentially confounding effect of obesity [31], body mass index (BMI) was also recorded. *C*. *difficile* infection, which has been associated with IBD [32], was recorded as the presence of a positive fecal *C*. *difficile* toxin B [33] within a week of the sample collection. Antibiotics have a significant effect on the microbiome [34] and all patients received preoperative surgical antibiotic prophylaxis (long-acting beta lactam 30 minutes before the initial incision was made) [35]. Patients diagnosed with *C*. *difficile* infections (predominantly colitis patients) were treated with oral vancomycin up until surgery. The few non-IBD (total <10) subjects from whom ileal research biopsies were collected during colonoscopy did not receive antibiotics prior to collection of the ileal biopsies for at least two months. Dietary information collected on the subjects revealed that none of the subjects were vegetarian and none of the subjects were on either elemental or polymeric enteral feedings [34]. Because there was no significant difference between the three institutions with respect to race and ethnicity (all predominantly non-Hispanic White/Caucasian), the Batch 2 patients were analyzed as a single cohort.

### IBD genotyping

Because of their relationship to Crohn’s disease phenotype, our analysis focused on the following single nucleotide polymorphisms (SNPs), which are implicated in microbial sensing, autophagy, endoplasmic reticulum stress, and/or Paneth cell dysfunction [6–10]: 1) NOD2 risk alleles (rs2066847, rs2066884, rs2066845, rs5743289) [9,36–41], ATG16L1 (rs2241880) [42–45], IRGM (rs13361189) [46–48], 2) CARD9 (rs10870077) [49,50], 3) XBP1 (rs35873774) [51], 4) ORMDL3 (rs2872507) [52]. The subjects were categorized as 1) homozygous for both non-risk alleles NR/NR), termed NR or 2) carrying at least one risk allele (R/NR, R/R), termed R. Four major NOD2 risk alleles were combined to form two composite categories: 1) NOD2^NR^, subjects harboring none of the four major risk alleles 2) NOD2^R^, subjects harboring at least one of the four major risk alleles (i.e., NOD2^R/NR^ + NOD2^R/R^). Illumina Immunochip genotyping using genomic DNA prepared from peripheral blood and/or tissue was performed on all of the subjects [53–55]. A subset of these patients had previously undergone genotyping by using the Sequenom MassArray System (Sequenom Inc., San Diego, CA) in the Washington University Sequenom Technology Core 3]. In the patients for which genotyping of the three major nonsynonymous NOD2 risk alleles, Leu1007fs (rs2066847, SNP13), R702W (rs2066884, SNP8) and G908R (rs2066845, SNP12), could not be assigned by Illumina Immunochip, genotyping for these SNPs was performed by Taqman Genotyping Assays (Life Technologies, Grand Island, NY, USA) as previously described [4]. For the ATG16L1 and IRGM genotypes, the value of a missing SNP was imputed from other tightly linked SNPs. For ATG16L1 the tightly linked SNP was rs12994997 and for IRGM genotype the SNPs were rs10065172 and rs11747270.

### 16S rRNA amplicon library construction and sequencing

Amplicons of the V3-V5 hypervariable regions of the bacterial 16S rRNA gene were sequenced using the 454 FLX Titanium Sequencing Platform and the same primers employed for characterizing the microbial communities in healthy individuals at different body sites including the gastrointestinal tract by the Human Microbiome Project [4, 56]. Library construction and sequencing for Batch 1 samples (2005–2010), was performed at the Genome Institute at Washington University-St. Louis. Library construction and sequencing for a small subset (n = 15) of Batch 1 samples and for all the Batch 2 (2010–2012) samples for all subjects recruited was performed in the Frank laboratory (UC Denver) and the sequencing was performed at The Centre for Applied Genomics at the Hospital for Sick Children in Toronto, Canada following the same standard operating procedures [4]. The variance in the relative abundances of phyla/subphyla taxa between duplicate libraries sequenced at the two different centers (n = 15) did not exceed the variance observed for duplicate libraries sequenced at a single center (n = 15). Clinical, genotyping, and sequencing data can be accessed through the dbGAP authorized access system (Request access to: phs000255.v2). In order to request access to any of the individual-level datasets within the controlled-access portions of the database, the Principal Investigator (PI) and the Signing Official (SO) at the investigator’s institution will need to co-sign a request for data access, which will be reviewed by an NIH Data Access Committee at the appropriate NIH Institute or Center (https://dbgap.ncbi.nlm.nih.gov/aa/wga.cgi?page=login).

Sequence reads were screened for basic quality defects by the software program BARTAB [57]. All sequences were checked for chimerism with Uchime (usearch6.0.203_i86linux32) [58] using the Schloss Silva references [59]. The filtered sequences were aligned and classified with SINA (1.2.11 using the 418,497 bacterial sequences in Silva 115NR99 as reference configured to yield Silva technology [60,61]. Operational taxonomic units (OTUs) at the genera level were produced by clustering sequences with identical taxonomic assignments. Relative abundances were calculated by dividing OTU counts were normalized between samples by dividing sequence counts by the total number of high-quality 16S sequences generated per sample to calculate the average relative abundance values shown in (**Table 2**). Phylum/subphyla level OTU tables were generated by collapsing lower level OTUs into higher-level categories corresponding to the categories used previously [4]: 1) *Actinobacteria*, 2) *Bacteroidetes*, 3) *Firmicutes/Clostridia/Ruminococcaceae* (family corresponding to Clostridia Group IV), 4) *Firmicutes/Clostridia/Lachnospiriceae* (family corresponding to Clostridia Group XIVa), 5) *Firmicutes/Clostridia/Other*, 6) *Firmicutes/Bacillus* (class), 7) *Firmicutes/Other*, 8) *Proteobacteria*, and 9) Other taxa.

**Table 2:** Comparison of the relative abundances of phyla/subphyla taxa between the disease unaffected and disease affected regions of resected ileum in ileal CD subjects undergoing initial ICR. The mean relative abundance ± standard deviation is shown for each bacterial category in Batch 1 (2005–2010) and Batch 2 (2010–2012) as well as the FDR for respectively macroscopic pathology (disease affected vs. disease unaffected) and the sample batch (Batch 1 Batch 2). A total of 101 disease affected and 111 disease unaffected samples were analyzed, of which 91 were paired samples.

	Disease affected		Disease unaffected		FDR	
	Batch 1	Batch 2	Batch 1	Batch 2	Path	Batch
*Actinobacteria*	0.028 ±0.055	0.006 ±0.011	0.049 ±0.088	0.009 ±0.016	0.092	<0.001
*Bacteroidetes*	0.314 ±0.272	0.447 ±0.255	0.292 ±0.273	0.356 ±0.278	0.346	0.165
*Firmicutes/Clostridia/* *Lachnospiraceae*	0.166 ±0.161	0.172 ±0.141	0.127 ±0.135	0.148 ±0.144	0.071	0.449
*Firmicutes/Clostridia/* *Ruminococcaceae*	0.028 ±0.039	0.045 ±0.074	0.021 ±0.031	0.036 ±0.08	0.023	0.473
*Firmicutes/Clostridia/* *Other*	0.059 ±0.121	0.056 ±0.127	0.058 ±0.136	0.061 ±0.141	0.631	0.449
*Firmicutes/Bacilli*	0.124 ±0.195	0.051 ±0.119	0.149 ±0.177)	0.088 ±0.177	0.071	<0.001
*Proteobacteria*	0.158 ±0.206	0.146 ±0.183	0.197 ±0.223	0.204 ±0.222	0.026	0.637

### Quantitative PCR for targeted bacterial subgroups

QPCR assays were performed using established primers for total bacteria (forward, 5’- GTG STG CAY GGY TGT CGT CA-3’ and reverse 5’- ACG TCR TCC MCA CCT TCC TC-3’) [62], *F*. *prausnitzii* (forward 5’-CCC TTC AGT GCC GCA GT-3’ and reverse 5’-GTC GCA GGA TGT CAA GAC-3’) [63], *C*. *coccoides-E*. *rectales* subgroup (forward, 5’–CGG TAC CTG ACT AAG C-3’and reverse 5’–AGT TT(C/T) ATT CTT GCG AAC G-3’) [63]. The log_2_ transformation of the relative abundance of each bacterial subgroup was measured by ΔCt = Ct (threshold cycle) _total bacteria_−Ct _subgroup_. All assays were carried out in triplicate and results averaged. Plasmid quantification standards were prepared from representative clones of the target organisms to insure that the assays were conducted within the linear range and with similar slopes [64].

### Statistical analysis

Patient samples with less than 100 total high-quality 16S rRNA sequence counts were excluded from the analysis. Alpha diversity indices (i.e., Chao1, Shannon complexity H) were calculated for each using the software package Explicet (v2.9.4, www.explicet.org) [65]. Beta diversity was calculated using the adonis function in the R vegan package as previously described [66,67] at the phylum/subphylum level, family and genus levels. This function uses a non-parametric multivariate analysis of variance test (PERMANOVA) [68], which was applied using Bray-Curtis, Morisita-Horn and Jaccard indices as distance measurements [69].

Because over-dispersion is often observed in microbiome sequence count data, negative binomial regression models [70, 71] were used to analyze the 16S rRNA sequence data. The OTUs (relative abundance ≥0.0001 and prevalence ≥0.01) were grouped primarily at the phylum/subphylum level as described above [4]. To identify taxa with significant differences in relative abundance between disease affected and disease unaffected regions of the resected ileum in ileal CD patients undergoing initial ICR, a generalized linear mixed model was used:

$$log(\mu_{ijk})=\beta_{i0k}+\beta_{1k}Pathology_{ij}+\beta_{2k}{Batch}_{i}+(log\;total\;coun{t)}_{ij}$$

$$\beta_{i0k}=b_{0k}+b_{ik}I\{Patient=i\}$$

$$Y_{ijk}∼NB(\mu_{ijk},\phi_{k})$$

Here Y*ijk* denotes the OTU *k*’s observed count for tissue *j* in patient *i* with μ_*ijk*_ being its mean. The symbol *ϕ_k_* is the dispersion parameter for the count of OTU *k*. The OTU *k* of patient *i* is associated with a random coefficient *b*_*ik*_ in order to assess paired disease affected and disease unaffected samples collected from the same patient *i*. The *Batch* variable refers to whether samples were collected in Batch 1 (2005–2010) or Batch 2 (2010–2012). The log total count of each patient sample is considered as an offset.

Negative binomial models were used to identify associations between OTUs and IBD phenotype (ileal CD, colitis without ileal disease, and control non-IBD), IBD genotype, and clinical co-variates (see **Table 1**).

For OTU *k*, the model is:

$$Y_{ik}∼NB(\mu_{ik},\phi_{k})$$

$$log(\mu_{ik})=\beta_{0k}+\beta_{1k}phenotype_{i}+\Sigma_{j}\alpha_{jk}Genotypes_{ij}+\Sigma_{l}\gamma_{lk}{covariates}_{il}+\beta_{2k}Batch_{i}$$

$$+interactions+(log\;total\;count{)}_{i}$$

*Y_ik_* is the observed counts for subject *i* on OTU *k*. The IBD genotypes and covariates in the model are listed in **Table 1.** The *Batch* variable refers to whether samples were collected in Batch 1 (2005–2010) or Batch 2 (2010–2012). The interactions included first-order interactions between phenotype, genotypes, clinical covariates and Batch. Stepwise variable selection based on Bayesian information criterion (BIC) was applied to generate the final model. The p value of coefficients in final model was subject to FDR correction [72], with the FDR threshold set at 0.05.

The relative abundances of *F*. *prausnitzii* and *C*. *coccoides-E*.*rectales* measured by qPCR were analyzed using a permutation-based linear regression model.

## Results

### Distribution of IBD phenotype, genotype, and clinical covariates in Batch 1 (2005–2010) and Batch 2 (2010–2012) samples

Samples were analyzed from 154 subjects in Batch 2 in addition to the 170 subjects in Batch 1 analyzed in our previous study [4]. Of the 324 total subjects, genotype, and clinical data were completed for 283 (87%) subjects. The distributions of IBD phenotype, genotype, and clinical covariates for the 283 of 324 subjects with complete datasets are summarized in **Table 1**. All of the Batch 1 samples and the majority (62–83%) of Batch 2 samples were collected at the Washington University–St. Louis medical center and the remaining subjects (17–38%) were recruited at the Stony Brook University and University of North Carolina medical centers (**Table 1**). At all three institutions, the race and ethnicity of the subjects were predominantly White/Caucasian. The proportion of subjects for each disease phenotype who had at least one of the following risk alleles: the NOD2 risk super allele (rs2066847, rs2066884, rs2066845, rs5743289), the ATG16L1T300A allele (rs2241880), the IRGM risk allele (rs13361189), the CARD9 risk allele (rs10870077), the XBP1 risk allele (rs35873774), and the ORMDL3 risk allele (rs2872507) are shown in **Table 1**. The high proportion of NOD2^R^ non-IBD subjects in Batch 2 reflects preferential selection of these individuals since our previous analysis indicated that NOD2 genotype was associated with shifts in ileal microbiome composition, independent of disease phenotype.

The clinical covariates for Batch 1 and Batch 2 samples (**Table 1**) revealed that the median duration of IBD prior to initial surgery was 5 years for both ileal CD and colitis subjects. As previously noted [4], ileal CD subjects were younger than non-IBD patients, and active smoking was less prevalent in colitis subjects. A higher proportion of the Batch 2 ileal CD and colitis subjects received anti-TNF alpha biologics at the time of surgery than Batch 1 subjects (See **Table 1**). A lower proportion of the Batch 2 colitis subjects had *C*. *difficile* infections immediately prior to surgery than the Batch 1 colitis patients.

### 16S rRNA sequencing analysis of ileal CD, colitis and non-IBD samples

**Fig 1:**
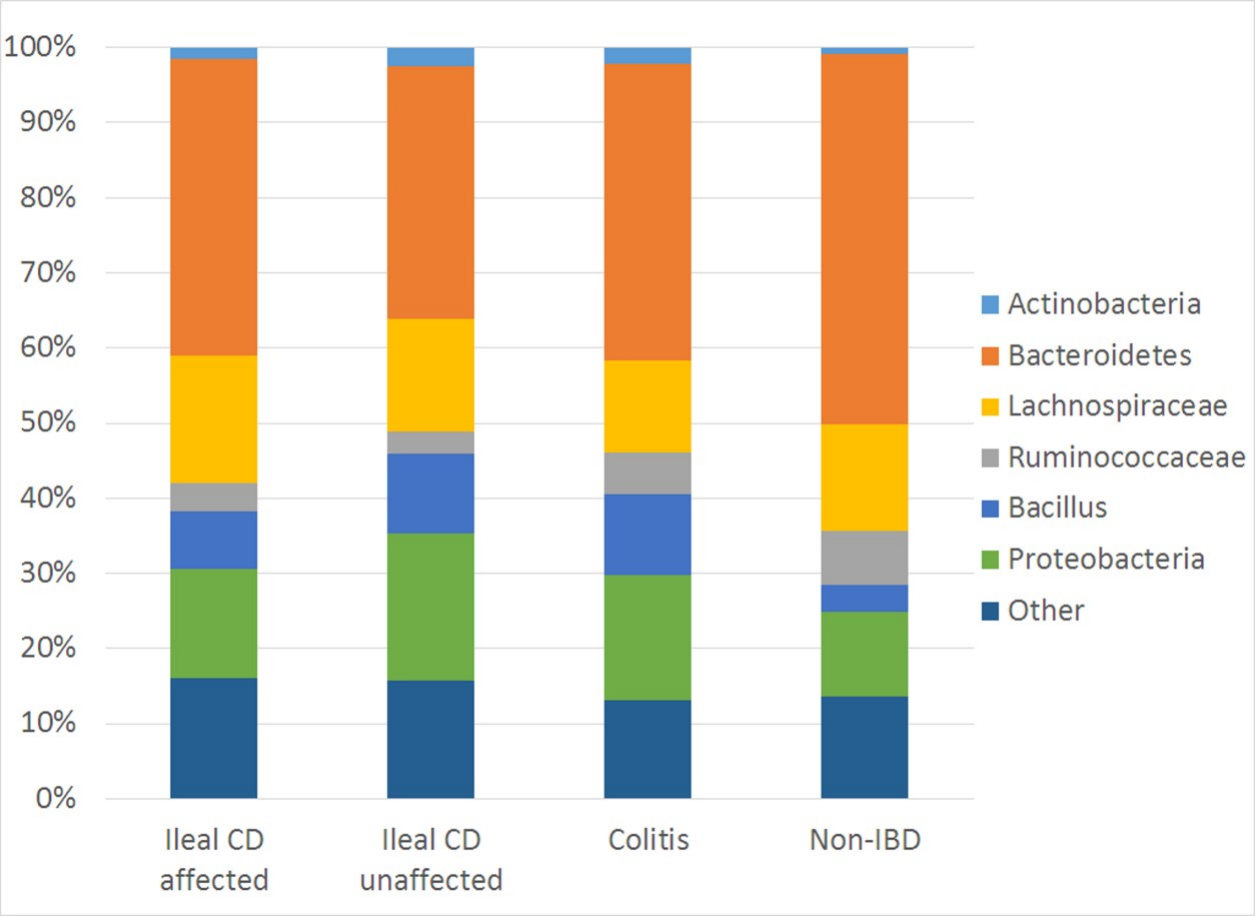
Phylum/Subphylum comparison of ileum-associated bacterial communities of ileal CD, colitis, and non-IBD samples. The average relative abundances of 6 of 8 phylum/subphylum bacterial category are shown for macroscopically disease affected and macroscopically disease unaffected samples, respectively from ileal CD, and from macroscopically disease unaffected samples from colitis and control subjects. In this figure “Other” includes Firmicutes/Clostridia/Other, Firmicutes/Other and all remaining taxa.

A total of 5,739,816 high-quality V3–V5 sequences (median 6024 reads/sample, IQR 2950–10314) were generated from the ileal specimens in this study. All libraries had a Goods coverage of ≥ 95% (median 98%, IQR 97.9–98.8%) at the rarefaction point of 500 sequences. All of the sequences were binned using an updated pipeline as described in *Methods*. Greater than 90% of the sequences could be binned into the following eight phylum/subphylum categories: 1) *Actinobacteria*, 2) *Bacteroidetes*, 3) *Firmicutes/Clostridia/Ruminococcaceae*, 4) *Firmicutes/Clostridia/Lachnospiraceae*, 5) *Firmicutes/Clostridia/Other*, 6) *Firmicutes/Bacillus*, *7) Firmicutes/Other*, 8) *Proteobacteri*a, (see **Fig 1**). The *Firmicutes* phyla were sub-divided between classes *Clostridia*, *Bacilli* and Firmicutes/Other. The Clostridia taxa were then further subdivided into *Clostridia/Ruminococcaceae* and *Clostridia/Lachnospiraceae* at the family level, corresponding to the Clostridium Group IV and XIVa phylum/subphylum categories designated when the Batch 1 samples were previously analyzed [4]. The remaining *Clostridia* OTUs were grouped as *Clostridia/Other*.

**Fig 2:**
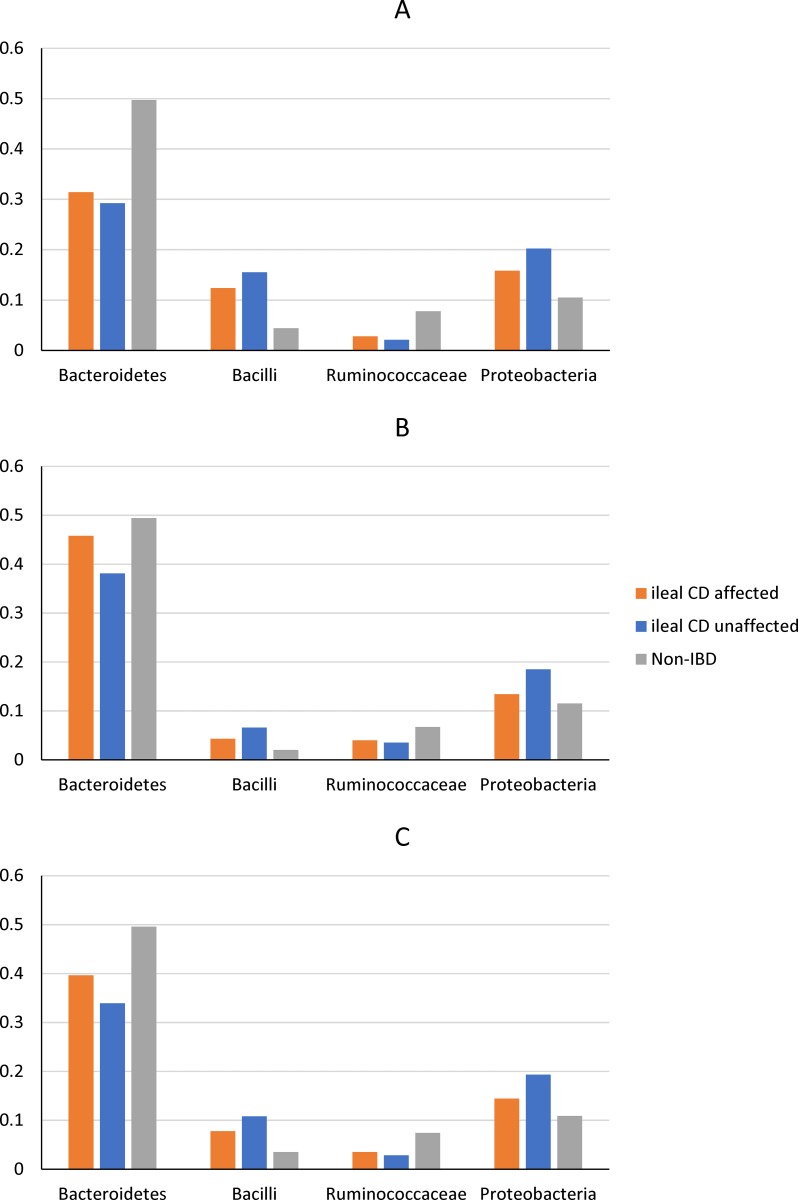
The mean relative abundances of selected phyla/subphyla groups between disease affected ileal samples from ileal CD subjects, disease unaffected ileal samples from ileal CD subjects and disease unaffected ileal samples from non-IBD subjects. A. Batch 1; B. Batch 2; C. Batch 1 and Batch 2 combined.

Because samples were collected from both the macroscopically disease unaffected region (proximal margin) and the disease affected region of the resected ileum, we first examined the within-subject differences in microbiota between disease unaffected and affected regions of the ileum. To this end, a generalized linear mixed effect negative binomial model was used to measure the effects of pathology (disease affected vs. disease unaffected) as well as sample batch on ileal microbiota binned at the phyla/subphyla level (see **Table 2**). The effect of pathology was significant for *Ruminococcaceae* (FDR = 0.023) and *Proteobacteria* (FDR = 0.026, see **Table 2**). As shown in **Fig 2** and **S1 Table**, the mean relative abundance of *Proteobacteria* was consistently higher in the disease unaffected regions than the adjacent disease affected regions from ileal CD subjects.

**Fig 3:**
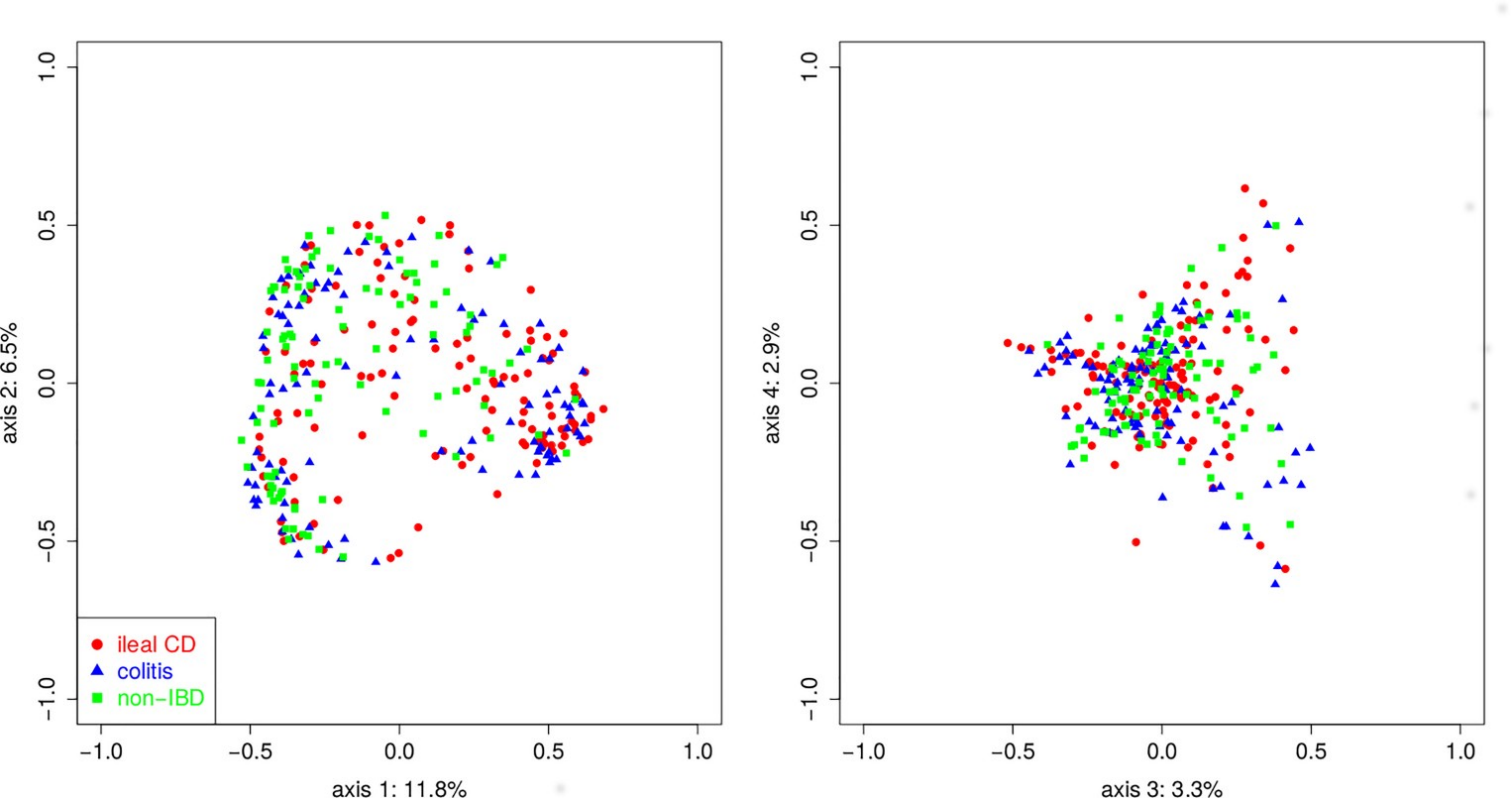
Principal coordinate analysis (PCoA) conducted on genus-level microbiome data using a dissimilarity matrix of Bray Curtis scores. The IBD phenotypes are color coded as follows: ileal CD (designated by red circle), colitis without ileal disease (designated by red triangle), non-IBD (designated by green square). The four largest dimensions (PC1, PC2, PC3, PC4) are shown and account for 11.8%, 6.5%, 3.3% and 2.9% of the differences, respectively.

No significant differences (p < 0.05) were detected in measures of alpha diversity (complexity measured by Shannon H or richness, measured by Chao1) of the ileal microbiome in ileal CD, colitis, and non-IBD disease unaffected samples or between disease unaffected and disease affected ileal CD samples. There were significant differences in beta diversity using three different dissimilarity indices, Bray-Curtis (p < 0.001, **Fig 3**), Jaccard (p < 0.001), and Morasita-Horn (p <0.001) of the ileal microbiome in disease unaffected regions between the three phenotypes. Furthermore, pairwise comparisons revealed significant differences between all three phenotypes (see **S2 Table**). While significant differences were detected for individual phyla/subphyla categories, particularly *Proteobacteria*, between disease unaffected and disease affected ileal CD samples, no significant difference in beta diversity was detected using any three of the dissimilarity indices.

### Integrated analysis of the effect of IBD phenotype, genotype and clinical covariates on phyla/subphyla bacterial categories

Because significant differences were detected between disease affected and disease unaffected samples from ileal CD subjects, analysis of the effects of IBD genotype (NOD2, ATG16L1, IRGM, CARD9, XBP1 and ORMDL3) on each phylum/subphylum category was restricted to only disease-unaffected samples from the three phenotypes (ileal CD, colitis, and non-IBD). Integrated analysis of the effects of the three IBD phenotypes, six IBD genotypes, 11 clinical covariates, sample batch, and all first order interactions, was conducted by building negative binomial models for each of the eight phyla/subphyla categories, using these factors as predictor variables (see **Table 3**).

**Table 3:** Negative binomial model results for IBD phenotype, genotype and clinical covariates at the phylum/subphylum level. Variables with significant effects are **bolded**.

*Actinobacteria*	Log fold change	p -value	FDR
**Main effects**^**1**^			
**ileal CD**	**2.055**	**<0.0001**	**<0.0001**
**Colitis**	**1.368**	**<0.0001**	**<0.0001**
**Duration IBD**	**-0.064**	**<0.0001**	**<0.0001**
**Batch**	**-1.194**	**<0.0001**	**<0.0001**
***+ C*. *difficile* fecal toxin**	**-2.878**	**0.0056**	**0.0083**
**Anti TNF alpha**	**-0.889**	**0.0015**	**0.0026**
Current smoker	-0.333	0.1081	0.1179
Age	0.001	0.9801	0.9801
**First order interactions**			
+ *C*. *difficile* fecal toxin*Current smoker	**2.806**	**0.0009**	**0.0017**
+ *C*. *difficile* fecal toxin *Age	**0.530**	**0.0124**	**0.0149**
Duration IBD * Anti TNF alpha	**0.008**	**0.0064**	**0.0083**
***Firmicutes/Clostridia/Lachnospiraceae***	**Log fold change**	**p -value**	**FDR**
**Main effects**			
**Immunomodulators**	**-0.347**	**0.0068**	**0.0068**
***Firmicutes/Clostridia/Ruminococcaceae***	**Log fold change**	**p -value**	**FDR**
**Main effects**			
**ileal CD**	**-0.989**	**0.0001**	**0.0002**
Colitis	-0.244	0.2714	0.2714
**Age**	**-0.017**	**0.0030**	**0.0032**
**Immunomodulators**	**1.879**	**0.0160**	**0.0224**
BMI	0.0282	0.0674	0.0787
**First order interactions**			
Immunomodulators * BMI	**-0.939**	**0.0012**	**0.0027**
***Firmicutes/Clostridia/Other***	**Log fold change**	**p -value**	**FDR**
**NOD2**^**R**^	**0.569**	**0.0028**	**0.0042**
**ORMDL3**^**R**^	**0.716**	**0.0001**	**0.0003**
**XBP1**^**R**^	**-1.000**	**0.0001**	**0.0003**
***+ C*. *difficile* fecal toxin**	**1.365**	**0.0001**	**0.0003**
**Steroids**	**0.606**	**0.0026**	**0.0042**
**Immunomodulators**	**-2.387**	**0.0027**	**0.0042**
**BMI**	**-0.0416**	**0.0065**	**0.0081**
Colon Cancer	-0.0384	0.8703	0.8703
Current smoking	-0.3546	0.2724	0.3143
**First order interactions**			
Current smoking * 5-ASA	1.9875	<0.0001	0.0002
Current smoking * Steroids	-1.287	0.006	0.0081
Immunomodulators * BMI	0.1074	0.0002	0.0005
***Firmicutes/Bacilli***	**Log fold change**	**p-value**	**FDR**
**Main effects**			
**ileal CD**	**1.7384**	**<0.0001**	**<0.0001**
**Colitis**	**1.2696**	**<0.0001**	**<0.0001**
**Batch**	**-0.9925**	**<0.0001**	**<0.0001**
**5-ASA**	**-0.8868**	**0.0049**	**0.0057**
Steroids	-0.3359	0.2659	0.2659
**First order interactions**			
5-ASA*Steroids	**1.4087**	0.0014	0.0019
***Proteobacteria***	**Log fold change**	**p-value**	**FDR**
**Main effects**			
**ileal CD**	**0.6767**	**<0.0001**	**0.0001**
**Colitis**	**0.5257**	**0.002**	**0.0023**
**NOD2**^**R**^	**-1.0788**	**0.0002**	**0.0003**
ORMDL3^R^	-0.2539	0.1433	0.1433
**First order interactions**			
**NOD2**^R^* ORMDL3^**R**^	**1.3856**	**<0.0001**	**0.0001**

The relative abundance of the *Actinobacteria* phylum was positively associated with both ileal CD and colitis phenotype and negatively associated with detection of *C*. *difficile* fecal toxin and anti-TNFα biologic use within 8 weeks of surgery. Significant first order interactions were observed for *C*. *difficile* fecal toxin b*age, *C*. *difficile* fecal toxin * current smoker, and IBD duration * anti-TNFα use.

The relative abundance of the *Firmicutes/Clostridia/Lachnospiraceae* (Clostridia Group XIVa) family was negatively associated with immunomodulatory use. The relative abundance of *C*. *coccoides-E*. *rectales* bacterial group determined by qPCR corresponds roughly to the relative abundance of the *Clostridia/Lachnospiraceae* family [4]. Univariate analysis did not detect a significant difference between NOD2^R^ and NOD2^NR^ ileal CD patients as previously reported for the Batch 1 samples [4]. A linear regression model (see **Table 4**) detected a positive association with the colitis phenotype. There was a trend towards a negative association between relative abundance of *the C*. *coccoides-E*. *rectales* group and immunomodulatory use and a significant association with the immunomodulator * *C*. *difficile* fecal toxin first order interactions.

**Table 4:** Linear regression results for disease phenotype, IBD genotype and additional clinical covariates (see Table 1) and the relative abundance of the *C*. *coccoides-E*. *rectales* bacterial subgroup and *F*. *prausnitzii*. Log fold change was determined by real time qPCR. Variables with significant effects were **bolded**.

*C*. *coccoides-E-rectales bacterial subgroup*	Log fold change	p-value	FDR
**Main effects**			
ileal CD	0.129	0.772	0.772
**Colitis**	**1.456**	**0.002**	**0.004**
Immunomodulators	-0.762	0.095	0.143
*C*. *difficile* fecal toxin	-1.08	0.290	0.347
**First order interactions**			
Immunomodulators* *C*. *difficile* fecal toxin	**4.71**	**0.001**	**0.004**
***Faecalibacterium prausnitzii***	**Log fold change**	**p-value**	**FDR**
**Main effects**			
**ileal CD**	**-1.691**	**0.001**	**0.001**
Colitis	-0.930	0.074	0.074

The relative abundance of the *Firmicutes/Clostridia/Ruminococcaceae* (Clostridia Group IV) family was strongly negatively associated with ileal CD phenotype and to a lesser extent with age, and positively associated with immunomodulator use. A significant first order interaction was observed for immunomodulators * BMI (see **Table 3**). At a more granular level, the relative abundance of the *Faecalibacterium* genus within the *Ruminococcaceae* family revealed significant negative associations with ileal CD phenotype (FDR = 0.0004) and age (FDR = 0.004), and significant positive associations with immunomodulatory use (FDR = 0.004) and BMI (FDR = 0.0006). In addition, a significant first order interaction was observed for immunomodulators * BMI (FDR = 0.0007). The negative association with ileal CD was further confirmed by measuring the relative abundance of *F*. *prausnitzii* by qPCR (FDR = 0.001, see **Table 4**). In addition, within the *Ruminococcaceae* (Clostridia Group IV) family, the relative abundance of the *Subdoligranulum* genus was also negative associated with the ileal CD phenotype (FDR < 0.0001) and colitis phenotype (FDR = 0.006).

Multiple associations, including IBD genotype (NOD2^R^, ORMDL3^R^, XBP1^R^), medications (steroids, immunomodulators), *C*. *difficile* fecal toxin and BMI were detected with the relative abundance of the remaining *Clostridia* taxa binned as *Firmicutes/Clostridia/Other* (see **Table 3**). Exploratory analyses did not detect associations between these three IBD genotypes with the predominant families binned within this group, such as *Clostridiales*/*Clostridiaceae* or *Clostridiales/Peptostreptococcaceae*.

The relative abundance of the *Firmicutes/Bacilli* class was positively associated with both ileal CD and colitis phenotype and negatively associated with 5-ASA (see **Table 3).** A significant first order interaction was observed for 5-ASA * Steroids. At a more granular level, similar associations with ileal CD (FDR = 0.04) and colitis (FDR = 0.02) phenotypes and negatively associated with 5-ASA (FDR = 0.003) were observed for the *Streptococcaceae* family, which is binned within this subphylum.

The relative abundance of the *Proteobacteria* phylum was positively associated with ileal CD and colitis phenotypes, but negatively associated with NOD2 genotype (see **Table 3**). Significant first order interactions were observed for NOD2^R^ * ORMDL3^R^ genotype and 5-ASA * anti-TNFα. Similar associations were observed for the relative abundance of the *Pseudomonadaceae* family and ileal CD phenotype (FDR <0.0001) and colitis phenotype (FDR = 0.0006). A similar association was observed between the relative abundance of the *Pseudomonas* genus and ileal CD phenotype (FDR <0.0001). In addition, significant associations were detected with XBP1^R^ (FDR = 0.0004) and with NOD2^R^*XBP1^R^ first order interactions (FDR = 0.005). Although the negative association between relative abundance of the *Enterobacteriaceae* family and NOD2^R^genotype did not reach significance (FDR = 0.15, FDR threshold = 0.05), a significant association was detected with first order NOD2^R^ * IRGM^R^ interaction. Finally, significant associations were detected between the *Enterobacteriaceae* family and ATG16L1^R^ genotype (FDR = 0.03), 5-ASA (FDR = 0.035), BMI (FDR = 0.001), and 5-ASA * BMI first order interactions (FDR = 0.01).

No significant effects were detected for the relative abundance of the remaining phyla/subphyla categories including the *Bacteroidetes* phylum.

### Microbial biomarkers of post-operative endoscopic recurrence in ileal CD subjects

Of the 124 ileal CD subjects included in this study, 61 subjects underwent postoperative ileoscopy of the neo-terminal ileum 6–12 months after surgery. The distribution of subjects for each Rutgeerts score were as follows: i0, n = 22; i1, n = 12; i2, n = 15; i3, n = 7; i4, n = 5. Therefore, 34 ileal CD subjects did not subsequently develop endoscopic recurrence (i0-i1) in the neo-terminal ileum, whereas the remaining 27 ileal CD subjects did subsequently develop endoscopic recurrence (i2-i4). Further analyses were conducted to compare the microbial composition of the disease unaffected region of the resected ileum in the 27 subjects who subsequently developed endoscopic recurrence with that of the 34 subjects who did not.

No difference in alpha- or beta-diversity at the phylum, family, or genus level, was detected in the disease unaffected specimens collected from the ileal CD subjects who did or did not subsequently develop recurrence. To identify potential microbial biomarkers of recurrence, univariate negative binomial analyses were conducted to identify taxa with significant differences in relative abundances between the ileal CD subjects who did or did not subsequently develop recurrence. No significant difference was observed when the bacteria were binned at the phyla/subphyla level. At the family level, the relative abundance of a number of bacterial taxa, including two potentially pathogenic families, *Pasteurellaceae* and *Mycobacteriaceae*, differed between ileal CD patients with and without recurrence (see **Table 5**). However, although the relative abundance of the *Ruminococcaceae* family was decreased in ileal CD patients who subsequently developed endoscopic recurrence compared to those who did not, the difference did not reach statistical significance. QPCR measurements of the relative abundance of *C*. *Coccoides-E*. *rectales* (i.e., members of the *Lachnospiraceae* family), and *F*. *prausnitzii* (i.e., an abundant species of the *Ruminococcaceae* family indicated significantly reduced levels of both taxa in patients with endoscopic recurrence (see **Table 4**). The difference between the qPCR and 16S rRNA sequence count data may reflect in part the looser correlation between qPCR and sequencing compared to the correlation between different sequencing platforms [73].

**Table 5:** Univariate negative binomial results (p-value and FDR) for endoscopic recurrence: family-level analysis. Only families with FDR corrected p-values <0.05 are included.

	Ileal CD		p-value	FDR
Increased in recurrence	Recurrence Mean ±std	No recurrence Mean ±std		
*Proteobacteria/Gammaproteobacteria/* *Pasteurellales/Pasteurellaceae*	0.0467 ±0.1581	0.0173 ±0.0664	1E-5	0.002
*Proteobacteria/Alphaproteobacteria/* *Rhodospirillales/ Acetobacteraceae*	0.0005 ± 0.1583	0.0000 ±0.0001	5E-5	0.004
*Firmicutes/Clostridia/Clostridiales/* *Other*	0.0354 ± 0.1405	0.0022 ± 0.0046	4E-4	0.016
*Actinobacteria/Corynebacteriales/* *Mycobacteriaceae*	0.0001 ±0.0003	0.0000 ±0.0003	0.001	0.026
**Decreased in recurrence**				
*Proteobacteria/Alphaproteobacteria/* *Rhizobiales/ Methylobacteriaceae*	0.0092 ± 0.023	0.0137 ± 0.0505	1E-4	0.006
*Proteobacteria/Betaproteobacteria/* *Burkholderiales/ Comamonadaceae*	0.0028 ± 0.0044	0.018 ± 0.0663	0.001	0.02
*Proteobacteria/Gammaproteobacteria/ Pseudomonadales/Moraxellaceae*	0.001 ± 0.002	0.0185 ± 0.0799	0.001	0.02
*Proteobacteria/Betaproteobacteria/* *Burkholderiales/ Alcaligenaceae*	0.004 ±0.005	0.018 ± 0.02	0.001	0.02
*Firmicutes/Erysipelotrichi/* *Erysipelotrichales/Erysipelotrichaceae*	0.0001 ± 0.0003	0.007 ± 0.030	0.002	0.03

## Discussion

The current studies report findings from an integrative approach to examine the relationships between host genetic factors, clinical factors, and dysbiosis in an expanded IBD patient dataset collected from three institutions with approximately double the number of subjects than was previously reported [4]. This study focused on ileal CD phenotypes and, to reduce heterogeneity, included two non-overlapping phenotypes without ileal CD: 1) non-IBD subjects and 2) colitis subjects without evidence of ileal disease. It is important to note however that the IBD genotype profiles associated with UC/indeterminate colitis, Crohn’s colitis and ileal CD/ileocolonic CD phenotypes represent a continuum [9]. Our analysis emphasizes samples taken from macroscopically disease-unaffected regions of the ileum, because we detected significant differences in ileal microbiota between samples obtained from adjacent disease-affected regions of the ileum, and included only samples from initial ileocolic resections. This is because increased reflux of colonic content into the neo-terminal ileum, would be anticipated after surgical removal of the ileocolic valve.

This study linking IBD genotype and clinical covariates to ileal mucosal samples represents one of the larger patient cohorts and is comparable in size to a previous study, which combined ileal mucosal biopsies from three institutions in Boston, Toronto and the Netherlands [55]. The V3-V5 pyrosequencing platform used in the current study, differs from the previous study which utilized the V4 Illumina sequencing [55]. The Illumina sequencing platform is generally conducted at a greater depth of sequencing (9,55,76–79), however Illumina sequencing read lengths (V1-V2, V3-V4, V4) are shorter than the pyrosequencing read length (V3-V5),. In the current study, significant differences in α-diversity were not detected between the three phenotypes, possibly because of the lower depth of sequencing using the pyrosequencing platform.

The dimensions of the linked data sets in the current study were reduced for integrative analysis by restricting the number of distinct non overlapping IBD phenotypes to 3, binning the taxa into 8 major phyla/subphyla categories, and by restricting the IBD loci to 6 that have previously been associated with innate immunity. The analysis was also restricted to macroscopically normal appearing disease unaffected ileum at the time of initial surgical resection, in contrast to the previous study where the ileal samples analyzed were more heterogeneous [55]. Our study is also unique in that detection of fecal *C*. *difficile* toxin (primarily in colitis subjects) was included as a potentially confounding co-variate. The rationale for including this co-variate is based on our previous study of Batch 1 subjects [4], and a more recent study analyzing the effect of FMT demonstrated marked dysbiosis associated with recurrent *C*. *difficile* infections in patients with and without ulcerative colitis compared to healthy subjects and compared to ulcerative colitis patients without *C*. *difficile* infections [74]. However it remains difficult to determine whether the marked dysbiosis is related to *C*. *difficile* infection or antibiotic treatment of the infection.

This expanded study confirmed our previous report [4] linking NOD2^R^ genotype, *C*. *difficile* infection, and IBD phenotype to ileal dysbiosis in Batch1 subjects alone, despite the addition of four additional genetic loci associated with defects in innate immunity (IRGM, CARD9, XBP1, and ORMDL3) to the analysis. In our previous study [3], NOD2^R^ genotype was linked to the relative abundance of the *Proteobacteria* phylum (p = 0.016, FDR = 0.07) independent of IBD phenotype. In this current expanded study, this association now reached significance (FDR = 0.0003). The observation that while ileal CD phenotype is positively associated with the relative abundance of *Proteobacteria* phylum in disease unaffected ileal mucosa, NOD2^R^ genotype is negatively associated is somewhat puzzling. It may be related to the observation that the mean relative abundance of *Proteobacteria* was consistently lower in disease affected ileal mucosa than in adjacent disease unaffected mucosa in ileal CD subjects. Both observations reinforce the concept that dysbiosis is detectable in macroscopically normal ileal mucosa in ileal CD subjects

Aggregate analysis at the phylum/subphylum level could mask the contribution of individual taxa within each of these groups, particularly if significant associations with phylogenetically related taxa exhibit opposing polarities. Identifying significant associations with bacterial subcategories at the family or genus level likely was limited by the reduced relative abundance of the individual taxa and the increased number of multiple comparisons. We are currently pursuing deeper sequencing and expanding the patient cohort to increase statistical power to detect differences in lower-level taxa. Nonetheless, at a more granular level, a possible effect of NOD2 genotype was detected through NOD2 * XBP1 first order interactions with the relative abundance of *Pseudomonas* genus, and through the NOD2*IRGM first order interaction with the relative abundance of the *Enterobacteriaceae* family (FDR <0.05). The NOD2*IRGM interaction may reflect, at least in part, direct interactions between the IRGM, NOD2, and ATG16L1 gene products, which have been reported to form a molecular complex that modulates autophagic reactions to microbial products [8]. NOD2^R^ genotype was not significantly associated with lower *Enterobacteriaceae* (FDR = 0.15) based on our threshold of FDR = 0.05 in the current study, but the results suggest a trend. In contrast, NOD2 risk dosage has been previously correlated with higher *Enterobacteriaceae* (FDR = 0.11, FDR threshold = 0.25) in ileal samples collected in two of three cohorts in the previous study (Boston and the Netherlands, n = 314) [55]. In summary, while both the current and the previous IBD genotype–ileal microbiota studies [55] detect associations between NOD2 risk alleles and Proteobacteria taxa, these associations had opposite polarities. This may relate to the analysis of only macroscopically disease unaffected ileal sample obtained from initial ileal resection in three relatively distinct IBD subphenotypes (ileal CD, colitis and non-IBD) in the current study.

NOD2 and other additional IBD genotypes (ORMDL3, XBP1) were also linked to the relative abundance of *Clostridia* spp. not binned into either the *Ruminococcaceae* or *Lachnospiraceae* families, but we were unable to attribute this association to any of the taxa within this bacterial category. In the expanded study we could not confirm the increased abundance of *C*. *coccoides–E*. *rectales* group and *F*. *prausnitzii* previously detected by PCR analysis in NOD2^R^ ileal CD subjects compared to NOD2^NR^ ileal CD subjects in Batch 1 alone [4]

A recent analysis of the effect of NOD2 homozygotes and compound heterozygotes compared to wild type NOD2 homozygotes in CD patients in clinical remission and in non-IBD controls detected no significant differences in the fecal microbiota [75]. This may be due in part to the smaller sample size, to the asymptomatic status of the CD patients, and to compositional differences between ileal microbiota and fecal microbiota [76–78].

Associations between ileal CD phenotype were confirmed with the decreased relative abundance of the *Ruminococcaceae* family and increased relative abundances of the *Actinobacteria* phylum and the *Firmicutes/Bacillus* class. These analyses also confirmed similar associations between the relative abundances of *F*. *prausntizii* within the *Ruminococcaceae* family, the *Corynebacteriaceae* family in the *Actinobacteria* phylum, and the *Streptococcaceae* family in the *Bacillus* class.

The current study has a larger or comparable cohort size compared with previous studies on microbial predictors of post-ICR endoscopic recurrence at the time of resection [17,22–25,79]. Q-PCR analysis of *F*. *prausnitzii* abundance confirmed that there was reduced abundance of this species in subjects that subsequently went on to develop endoscopic recurrence at 6–12 months compared to those that did not develop recurrence. In addition exploratory analysis identified two potentially pathogenic taxa, *Pasteurellaceae* and *Mycobacteriaceae*, with increased abundance in subjects that subsequently developed endoscopic recurrence compared to those that did not. Atypical mycobacteria have been implicated in the pathogenesis of CD [80]. The abundance of *Pasteurellaceae* is increased in fecal microbiota collected from treatment-naïve new-onset CD subjects compared to non-IBD subjects [21]. Somewhat puzzling is a report from a recent study [78] that the abundance of *Pasteurellaceae* is *increased* in the resected ileum collected from patients *that remain in remission* compared to those that have endoscopic recurrence. It remains to be determined whether the taxa associated with endoscopic recurrence simply reflect subtle alterations in the local environment, such as changes in pH or oxidation, or whether they play a causal role in promoting recurrent inflammation in the peri-anastomotic neo-terminal ileum.

Our study likely was limited by the batch effect observed, most notably for the relative abundance of the Actinobacteria phylum and the Firmicutes/Bacilli class between Batch 1 (2005–2010) and Batch 2 (2010–2012) cohorts. Several differences between the two patient sample cohorts, Batch 1 and 2, could contribute to this effect. Despite the use of the same primers for library construction and the same pyrosequencing platforms, which generated concordant results in a preliminary duplicate analysis of a small subset of samples, some systematic technical errors may have contributed to batch effects. Another factor could be related to demographic differences between Batch 1 and Batch 2 patients, which included a higher proportion of NOD2^R^ non-IBD patients, a higher proportion of patients treated with anti-TNFα biologics, and a lower proportion of patients treated with 5-ASA medications in Batch 2 compared to Batch 1 (see **Table 1**) This study was also limited with respect to assessing the effect of antibiotics, because all of the patient received antibiotics prior to the collection of the samples during surgical resection except <10 non IBD subjects, who contributed endoscopic biopsies in Batch 2, where they received no antibiotics.

In summary, the results of this integrative analysis of a large number of uniformly curated samples, confirm a significant effect of IBD phenotype, *C*. *difficile* infection, and NOD2 genotype on ileum-associated microbiota. Furthermore, we present data demonstrating that additional IBD-related genotypes, specifically alleles of ORMDL3 and XBP1, are associated with changes in the ileal microbiome at the phyla/subphyla level, particularly Proteobacteria, either as direct effects or through interactions with NOD2 or other clinical variables. As we continue to expand our systematic accrual of subjects, further associations between host, environment, and microbial factors with ileal CD phenotype and clinical outcome will emerge and help delineate the complex etiology of this disease.

## Supporting information

S1 Table Comparison of relative abundances of Bacteroidetes, Bacilli, Ruminococcaceae and Proteobacteria phyla/subphyla categories in disease affected and disease unaffected ileal CD and disease unaffected non-IBD samples for Batch 1, Batch 2, and Batch 1 + 2 combined. (DOCX)

S2 Table Pairwise comparisons of beta-diversity between the three phenotypes, ileal CD, colitis, and non-IBD. (DOCX)
