# Supplementary material for: Influence of Crohn’s disease related polymorphisms in innate immune function on ileal microbiome
Source: PLoS One. 2019 Feb 28;14(2):e0213108. doi: 10.1371/journal.pone.0213108 (PMC6395037; doi:10.1371/journal.pone.0213108)
Supplement: S1 Table — (DOCX) [file pone.0213108.s001.docx]

**S1 Table. Comparison of relative abundances of Bacteroidetes, Bacilli, Ruminococcaceae and Proteobacteria phyla/subphyla categories in disease affected and disease unaffected ileal CD and disease unaffected non-IBD samples for Batch 1, Batch 2, and Batch 1 + 2 combined.** The mean relative abundances ± standard deviations (std dev) are shown.

|  | **Disease affected ileal CD** | **Disease unaffected ileal CD** | **Disease unaffected non-IBD** |
| --- | --- | --- | --- |
| ***Batch 1*** |  |  |  |
| *Bacteriodetes* | 0.31 ± 0.27 | 0.29 ± 0.27 | 0.50 ± 0.19 |
| *Bacilli* | 0.12 ± 0.20 | 0.16 ± 0.18 | 0.04 ± 0.08 |
| *Ruminococcaceae* | 0.03 ± 0.04 | 0.02 ± 0.03 | 0.08 ± 0.07 |
| *Proteobacteria* | 0.16 ±0.21 | 0.20 ± 0.24 | 0.10 ± 0.17 |
| ***Batch 2*** |  |  |  |
| *Bacteriodetes* | 0.46 ± 0.25 | 0.38 ± 0.26 | 0.49 ± 0.22 |
| *Bacilli* | 0.04 ± 0.08 | 0.07 ± 0.15 | 0.02 ± 0.05 |
| *Ruminococcaceae* | 0.04 ± 0.07 | 0.04 ± 0.07 | 0.07 ± 0.08 |
| *Proteobacteria* | 0.13 ± 0.17 | 0.18 ±0.20 | 0.12 ± 0.15 |
| ***Batch 1 ± Batch 2*** |  |  |  |
| *Bacteriodetes* | 0.40 | 0.34 | 0.50 |
| *Bacilli* | 0.08 | 0.11 | 0.04 |
| *Ruminococcaceae* | 0.03 | 0.03 | 0.07 |
| *Proteobacteria* | 0.14 | 0.19 | 0.11 |
