# Supplementary material for: Influence of Crohn’s disease related polymorphisms in innate immune function on ileal microbiome
Source: PLoS One. 2019 Feb 28;14(2):e0213108. doi: 10.1371/journal.pone.0213108 (PMC6395037; doi:10.1371/journal.pone.0213108)
Supplement: S2 Table — (DOCX) [file pone.0213108.s002.docx]

**S2 Table. Pairwise comparisons of beta-diversity between the three phenotypes, ileal CD, colitis, and non-IBD.** Results are p-values for pairwise PERMANOVA tests performed with the indicated beta-diversity index.

| Dissimilarity Indices | ileal CD vs. non-IBD | colitis vs. non-IBD | ileal CD vs. colitis |
| --- | --- | --- | --- |
| Bray-Curtis | 0.001 | 0.001 | 0.007 |
| Morasita-Horn | 0.001 | 0.001 | 0.028 |
| Jaccard | 0.001 | 0.001 | 0.003 |
